# Antimicrobial Activity of Host-Derived Lipids

**DOI:** 10.3390/antibiotics9020075

**Published:** 2020-02-11

**Authors:** Carol L. Fischer

**Affiliations:** Biology Department, Waldorf University, Forest City, IA 50436, USA; carol.fischer@waldorf.edu; Tel.: +1-319-530-4316

**Keywords:** antimicrobial lipids, sphingoid bases, fatty acids, antibacterial, innate immunity

## Abstract

Host-derived lipids are increasingly recognized as antimicrobial molecules that function in innate immune activities along with antimicrobial peptides. Sphingoid bases and fatty acids found on the skin, in saliva and other body fluids, and on all mucosal surfaces, including oral mucosa, exhibit antimicrobial activity against a variety of Gram positive and Gram negative bacteria, viruses, and fungi, and reduce inflammation in animal models. Multiple studies demonstrate that the antimicrobial activity of lipids is both specific and selective. There are indications that the site of action of antimicrobial fatty acids is the bacterial membrane, while the long-chain bases may inhibit cell wall synthesis as well as interacting with bacterial membranes. Research in this area, although still sporadic, has slowly increased in the last few decades; however, we still have much to learn about antimicrobial lipid mechanisms of activity and their potential use in novel drugs or topical treatments. One important potential benefit for the use of innate antimicrobial lipids (AMLs) as antimicrobial agents is the decreased likelihood side effects with treatment. Multiple studies report that endogenous AML treatments do not induce damage to cells or tissues, often decrease inflammation, and are active against biofilms. The present review summarizes the history of antimicrobial lipids from the skin surface, including both fatty acids and sphingoid bases, in multiple human body systems and summarizes their relative activity against various microorganisms. The range of antibacterial activities of lipids present at the skin surface and in saliva is presented. Some observations relevant to mechanisms of actions are discussed, but are largely still unknown. Multiple recent studies examine the therapeutic and prophylactic uses of AMLs. Although these lipids have been repeatedly demonstrated to act as innate effector molecules, they are not yet widely accepted as such. These compiled data further support fatty acid and sphingoid base inclusion as innate effector molecules.

## 1. Introduction

Skin and mucosal membranes function as a physical boundary between humans and their environments. Keratinocytes and associated skin or mucosal glands further contribute to this natural barrier through the production and secretion of a diverse array of noncellular antimicrobial products. These specific and nonspecific innate immune factors include simple inorganic molecules (e.g., peroxidases, nitric oxide), complement proteins, C-reactive protein, and a variety of antimicrobial peptides (AMPs) [1,2] and enzymes (e.g., defensins, lysozyme, lactoferrin) [3]. More recently, multiple endogenous lipid groups, present on the skin, in saliva, and on mucosal surfaces, have been described based on their ability to act as antimicrobial agents [3,4,5]. The hydrophobic nature of these antimicrobial lipids (AML) contributes to impermeability of the skin, preventing water loss while naturally impeding entrance of microorganisms and most toxins, while the antimicrobial nature of these natural lipids protects the body from invasion by many pathogens and opportunistic bacteria. Fatty acids and sphingoid bases are endogenous components of human skin and mucosal surfaces [6,7,8,9,10,11,12,13], cerumen [14], saliva [15], breast milk [16,17,18,19,20], and blood [21] and demonstrate antibacterial, antifungal, antiviral, and antiparasitic activity [16,22]. AMLs arise mainly from epidermal and sebaceous sources, where their antimicrobial activity appears to be both lipid-specific and microorganism-specific, exhibiting differential activity against bacteria, viruses, fungi, and parasites.

Although multiple studies have focused on the antimicrobial activity of fatty acids, the antimicrobial activity of sphingoid bases is still relatively unknown. Both fatty acids and sphingoid bases exhibit dose-dependent and specific antimicrobial activity, lending credence to idea that both serve an important innate function in the oral cavity, on the skin, and in other body fluids. Although multiple studies examined below demonstrate the innate activity of fatty acids and sphingoid bases, these important innate lipids are not yet broadly accepted as innate molecules. The main aims of this review are to tell the story of lipids as antimicrobial agents, demonstrate the broad antimicrobial activity of both fatty acids and sphingoid bases as demonstrated across multiple studies, discuss recent development of AMLs as therapeutic and anaphylactic treatments, and further support their inclusion as innate immune molecules. 

## 2. AML Sources

**Figure 1:**
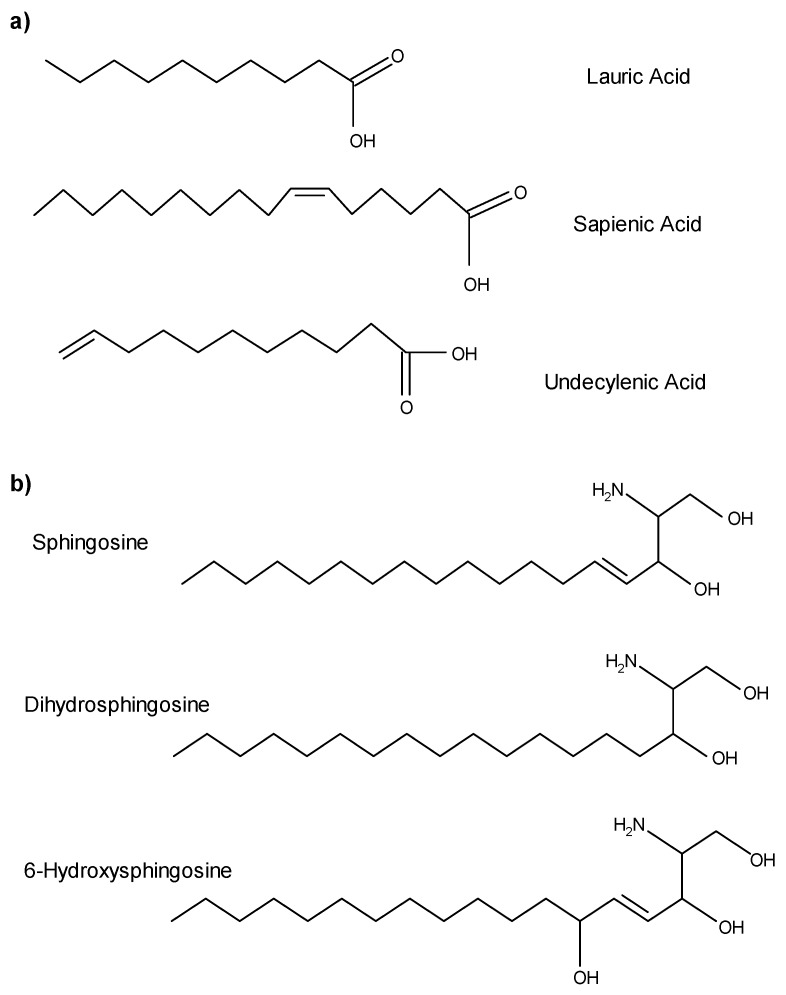
The chemical structures of antimicrobial lipids (AMLs) found on the surface of the skin and oral mucosa. (**a**) Sapienic acid is the major free fatty acid derived from sebum and lauric acid is the minor fatty acid derivative of sebum. Fatty acids demonstrating antimicrobial activity are generally 12–18 carbons and activity quickly decreases with either longer or shorter chains. Undecylenic acid is derived from sweat and also present on the skin surface. (**b**) Free sphingoid bases are derived from the stratum corneum, liberated via ceramidases, and exhibit broad antimicrobial activity.

Sebaceous glands produce triglycerides, wax monoesters, and squalene [23,24], and are the main source of antimicrobial fatty acids [25]. Triglycerides are synthesized in maturing sebocytes and released along with sebum into the follicular canal. As sebum flows through the follicular canal, triglycerides are hydrolyzed, releasing free fatty acids. Because sebaceous follicles are found within all nonglabrous epithelium, fatty acids are found throughout most regions of human skin, including all mucosal epithelium, the vermilion border of the lips, and the lining of the ear canals. Sebaceous fatty acids (Figure 1a) are amphipathic hydrocarbon chain molecules with a range of seven to 22 carbons and containing a terminal carboxylic acid group. Fatty acids differ in the number and placement of double bonds. Lauric acid (C12:0), a minor component of sebum, is a straight-chain, saturated, 12-carbon fatty acid. Sapienic acid (C16:1Δ6) is the most abundant fatty acid in the sebum of *Homo sapiens,* as evidenced by its name coined by Downing [26]. This 16-carbon straight-chain fatty acid has a cis double bond at carbon six which causes the molecule to twist, shrinking it to about the same physical size as a 14-carbon fatty acid. Of all the sebaceous fatty acids, sapienic acid and lauric acid are differentially active against a wide range of potentially pathogenic Gram positive bacteria and some yeasts. 

Because sebaceous follicles are associated with the major salivary glands, saliva also contains antimicrobial free fatty acids of sebaceous origin [15]. The total lipid fraction of saliva is predominantly neutral lipids, including free fatty acids, cholesterol, cholesterol esters, monoglycerides, diglycerides, and triglycerides [27,28,29] but also includes squalene and wax esters [15], which are biochemical markers of human sebum (Table 1). Undecylenic acid, a natural component of sweat, is also active against a variety of viruses and fungi [30] and this 11-carbon monounsaturated fatty acid is the active ingredient of many over-the-counter anti-fungal treatments.

Sphingoid bases are long chain amino alcohols consisting of a hydrocarbon chain, hydroxyl groups, and an amine group. Sphingoid bases vary in carbon chain length, degree of saturation and number of hydroxyl groups present. Whether they are found on the skin, on mucosal surfaces, or within the oral cavity, these amphipathic molecules are derived from lipids synthesized in the viable portion of epithelium. As epidermal keratinocytes move toward the surface, they differentiate and accumulate lipids (e.g., phospholipids, glycosylceramides, ceramides, sterols, sterol esters, and cholesterol) in lamellar bodies, which are extruded into intercellular spaces. Free sphingoid bases are not actually found in sebum, but hydrolytic enzymes present on the skin convert glucosylceramides and sphingomyelin into ceramides [32]. Finally, ceramidases, present in the keratinized regions of the skin, liberate sphingoid bases and free fatty acids from ceramides [33]. It is important to note that the fatty acids generated within epithelium are primarily long straight-chain fatty acids 20–28 carbons in length, with no demonstrated antimicrobial activity [6,34]; therefore, they should not be confused with antimicrobial fatty acids of sebaceous origin. Reported epithelial sphingoid bases with antimicrobial activity include sphingosine, dihydrosphingosine, (sphinganine), and 6-hydroxysphingosine (Figure 1b). Wertz and Downing [35] report varying concentrations of sphingosine and dihydrosphingosine between the inner and outer epithelial surfaces of human stratum corneum, which contributes to a distinct concentration gradient with higher sphingoid base concentrations in the stratum corneum and superficial layers of non-keratinizing regions. The antimicrobial activity of phytosphingosine, another common sphingoid base, is well documented [36,37,38,39,40]; however, even though phytosphingosine is present in some ceramides, free phytosphingosine has not been detected in human skin [41]. Although lipid concentrations of sphingoid bases likely vary between the skin, saliva, and mucosal membranes, and even across regions of epidermis and epithelia, the antimicrobial activity of lipids found in each location is likely similar. Sphingosine, dihydrosphingosine, and phytosphingosine have a broader range of activity against bacteria and yeast.

## 3. Fatty Acids as AMLs

In the 1940s, Burtenshaw provided some of the earliest evidence that skin lipids could act as antimicrobial agents when he demonstrated that lipids extracted from human skin surface scrapings killed *Staphylococcus aureus* [42]. He proposed that the main lipid fraction exhibiting antimicrobial activity was fatty acids and then repeated his studies with fatty acid extracts from hair, nails, and cerumen [43]. In the next few years, studies by Weitkamp, Smiljanic, and Rothman [44,45,46] examined the antimicrobial activity of free fatty acids. Initially, these investigators observed that increased sebum production at the onset of puberty corresponded with development of immunity to *tinea capitis* (ringworm of the scalp), a frequent recurrent fungal infection of infants and young children [44,46]. When they extracted and fractionated lipids from barber shop sweepings, they found that the fatty acid components of the lipid fraction were fungicidal to *Microsporum audouinii*, the causative agent of ringworm of the scalp [45]. The active fatty acids extracted from hair lipid fractions were ethanoic acid (C7:0), pelargonic acid (C9:0), undecylenic acid (C11:0), and tridecylic acid (C13:0). This was the first record of isolated short chain fatty acids from human samples and the first finding that these short, odd-carbon chain fatty acids possessed unique fungistatic capabilities. This finding clarified adult immunity to *tinea capitis* as changes in sebum production occurring during puberty. Much later, undecylenic acid, a related fatty acid from sweat, was discovered to exhibit similar antifungal activity against *M. audouinii, Epidermophyton inguinale, Candida albicans, Trichophyton rubrum,* and *Trichophyton mentagrophytes,* demonstrating its role in maintaining a healthy balance of vaginal, oral, and intestinal flora [30]. 

Following the novel studies by Burtenshaw, Weitkamp, Smiljanic, and Rothman [42,43,44,45,46], interest in the antimicrobial properties of fatty acids remained low and very few studies were completed in this area for several decades. Between 1972 and 1977, Kabara et al., published a series of papers testing over 40 fatty acids and their derivatives against a range of bacteria [47,48,49,50,51,52,53,54]. This research group found that C12 lipids such as lauric acid were the most potent against Gram positive bacteria but were not active against Gram negative bacteria. It was speculated that the differential activity against Gram positive and Gram negative bacteria could be due to the complexity and differences in their cell walls [54]. Differences in cell wall composition may allow better access to the bacterial membrane in Gram positive compared to Gram negative bacteria; however, a specific mechanism is unknown. Overall, lauric acid and sapienic acid are among the most active of the sebaceous fatty acids (see the summary of antimicrobial activity in Table 2) and both are active against a wide range of Gram positive bacteria [3,55]. Bergsson et al. [56] treated *S. aureus* with various fatty acids, including capric acid, a 10-carbon saturated fatty acid, and demonstrated subsequent disintegration of the bacterial membrane – but the cell wall appeared to remain intact. After several studies, Bergsson [57] specifically noted the specificity of fatty acids and monoglyceride (monoester formed from lauric acid and glycerol) activity against various bacteria. 

Fatty acids have also been tested against multiple oral bacterial species, some of which are active against both Gram negative and Gram positive bacteria (Table 2). Lauric acid and sapienic acid are active against *Aggregatibacter actinomycetemcomitans, Streptococcus mutans, Streptococcus gordonii, Streptococcus sanguinis, Fusobacterium nucleatum,* and *Porphyromonas gingivalis* [37,38,58]. Sapienic acid is more effective than lauric acid against all oral bacteria tested, including *P. gingivalis* and *F. nucleatum* [37,38] *. P. gingivalis* rapidly takes up sapienic acid and induces upregulation of proteins involved in multiple cellular processes, including lipid metabolism, and killing *P. gingivalis* in less than six minutes [37,59]. Scanning and transmission micrographs of treated and untreated bacteria demonstrate that sapienic acid also triggers creation of pores in the *P. gingivalis* cell membrane, flocculation of intracellular contents, alteration of the cellular membrane, and lysis of cells. In another study, total sebum lipids caused a 4- to 5-fold log reduction of *F. nucleatum* and *Streptococcus salivarius* [60]. The activity of sapienic acid and lauric acid against multiple Gram negative oral bacteria such as *A. actinomycetemcomitans, F. nucleatum, and P. gingivalis* is interesting, given that fatty acids are generally not active against Gram negative species. Based on the variance in activity seen in various bacteria-lipid combinations, it is possible that this can be contributed to variances in bacterial membrane lipids; however, no mechanism has yet been elucidated. 

Innate free fatty acids are also active against opportunistic bacteria on the skin. Huang, et al. [61] compared the activity of lauric acid, naturally present in skin [8,24], with capric acid, not endogenous to skin, against *Proprionibacterium acnes in vitro,* and *in vivo,* injecting bacteria and various treatments into mouse ears. Lauric acid activity was stronger than capric acid, although both reduced inflammation triggered by infection. Of particular interest was their finding that injection of lauric or capric acid alone did not produce any apparent irritation (e.g., swelling, redness, etc.) or induce inflammation, as indicated by the relative lack of inflammatory cytokines in the tissue. The anti-inflammatory properties of endogenous fatty acids is supported by the finding that various isomers of C16:1, including sapienic acid, are produced by human and murine phagocytic cells, and exhibit anti-inflammatory activity when added to cells stimulated with bacterial lipopolysaccharide [62]. In another pilot study examining the use of lauric acid as an antiseptic treatment for burn wounds [63], lauric acid was mixed with ointment bases and then applied to burnt skin collected during surgery. These samples were then treated with various bacteria, including *S. aureues* and *Escherichia coli.* All of the ointment base-lauric acid mixtures containing at least 20% lauric acid by weight, except for hydrophilic Vaseline base, exhibited pronounced inhibition of bacterial growth. The anti-inflammatory activity of endogenous fatty acids combined with the lack of side effects with their use favors the expansion of AML use in the development of new treatments and prophylactic agents.

Recent studies also highlight the role of host-derived AMLs in innate protection against gastrointestinal infections of adults and neonates. One very interesting study by Isaacs and colleagues [19] outlined the important protective value of milk fat against intestinal infection. This study demonstrated that the triglyceride content of milk is hydrolyzed by lipases within the gastrointestinal tract, releasing fatty acids and monoglycerides which are active against a variety of bacteria and viruses [18,19,20,64]. This information was later expanded by another group to include *Giardia lamblia* [16]. Lipids present in human breast milk are also hydrolyzed by lipases in the gastrointestinal tract of infants, releasing fatty acids that are active against several opportunistic bacteria of the gastrointestinal tract, including *E. coli, Campylobacter jejuni, Listeria monocytogenes,* and *Salmonella enterica* [17,18,19,20,65,66,67] 

More recently, lauric acid demonstrated effective, and dose-dependent activity in prevention of *Clostridium difficile* infections (CDI) in mouse CDI models [68]. Mice were administered daily high or low lauric acid doses orogastrically for one week prior to infection with *C. difficile,* followed by one dose after exposure to *C. difficile*. Lauric acid treatment prior to infection had some protective effects, but mice that were given high doses displayed healthier colons, less weight loss, and more normal stools than those administered low doses. This study also confirmed the damage of cellular membrane when cells were treated with even sub-lethal doses of lauric acid. 

AMLs also exhibit antiviral activities (Table 2). Phosphatidylinositol and palmitoyl-oleoyl-phosphatidylglycerol, both minor components of pulmonary surfactant [10], act as protective molecules against respiratory viruses. Palmitoyl-oleoyl-phosphatidylglycerol exerts activity against influenza A virus (IAV) by binding to viral particles, which prevents IAV infection of cells [69]. Phosphatidylinositol is a strong antiviral agent against respiratory syncytial virus both in vitro and in vivo [70]. A variety of fatty acids and monoglycerides are active against herpes simplex virus (HSV) and this antiviral activity becomes even more potent in increasingly acidic environments [71]. It is unknown whether this increased activity is due to lipid biochemistry or because HSV becomes more sensitive at lower pH levels.

## 4. Sphingoid Bases as AMLs

Although most studies focused on fatty acids as the major AMLs, epidermally-derived lipids, the sphingoid bases (long-chain bases), were added to the growing list of AMLs in 1988 with a study of the antimicrobial activity of human stratum corneum lipids [78]. This study was the first to demonstrate the significant anti-staphylococcal activity of polar lipids and glycosphingolipids. Shortly thereafter, Bibel, et al. [79,80,81,82,83] published a series of papers expanding the antimicrobial activity of sphingoid bases. These studies outlined the broad antimicrobial activity of sphingosine and dihydrosphingosine (sphinganine) against a variety of Gram positive and Gram negative bacteria and *C. albicans.* Many later studies confirmed the antimicrobial activity of sphingoid bases (see Table 3 for an overview of the antimicrobial activity of the innate sphingoid bases). Transmission electron micrographs of sphingoid base treated *S. aureus* showed ultrastructural damage similar to antibiotic treatment, including lesions of the cell wall, membrane evaginations, and leakage of the cellular contents. L-forms of *S. aureus* (lacking cell walls) are relatively resistant to the lethal effects of sphinganine, which suggests a specific action against the bacterial cell wall [82]. Importantly, significant differences in the antimicrobial activity among two sphinganine isomers, D,L-erythrosphinganine and D,L-threosphinganine, also supports the specificity of lipid treatments [80]. 

Sphingoid bases are also broadly active against oral bacteria (Table 3). Fischer and colleagues [36,37,59] tested sphingoid bases, sphingosine, dihydrosphingosine, and phytosphingosine, against a wide variety of Gram positive and Gram negative bacteria, including *E. coli, S. aureus, S. sanguinis, Streptococcus mitis, Corynebacterium striatum, Corynebacterium jeikeium, A. actinomycetemcomitans, F. nucleatum,* and *P. gingivalis* using minimum inhibitory concentrations (MIC), minimum bactericidal concentrations, and kill kinetics assays. Sphingoid bases were widely active against Gram positive bacteria and some Gram negative oral species of bacteria with variable and specific activity. The sphingoid bases were taken up by *S. aureus* and *E. coli,* which induced extracellular and intracellular morphological damage as judged by scanning and transmission electron microscopy [36]. Most interesting in these studies were the activities of multiple AMPs against oral bacteria [37,38]. *P. gingivalis* was killed by very low levels of sphingosine (0.2 ± 0.8 µg/mL), phytosphingosine (0.8 ± 0.3 µg/mL), and dihydrosphingosine (0.4 ± 0.2 µg/mL) at very low concentrations [37]. 

Recent studies demonstrate the possible use of sphingosine to prevent harmful bacterial biofilm formation on medical equipment. Biofilm-forming bacteria account for 65–85% of human infections [84], many of which can form on medical equipment, orthopedic implants, catheters, pacemakers, contact lenses, prosthetic joints, and many other medical devices. Because of the ability of biofilms to remain on inanimate surfaces for long periods of time [85], staphylococcal infections, especially *S. aureus* infections, are the leading cause of nosocomial infections in the United States [86,87], many of which arise from contaminated equipment and other inanimate surfaces [88]. The formation of biofilms confers considerable resistance against host immune responses, antibiotics, and antiseptics [84,85]. Resistance mechanisms are largely unknown but are partly due to the inability of most agents to fully penetrate biofilm matrix. Sphingosine- and phytosphingosine-coating of plastic medical devices, such as endotracheal tubes, provides protection against adherence and infection of Gram positive and Gram negative bacteria associated with ventilator-associated pneumonia [89]. These treatments prevent growth of *Pseudomonas aeruginosa, Acinetobacter baumannii,* and methicillin-resistant *S. aureus,* and are 90%-99% bactericidal against these bacteria, even in biofilms. In this study, the sphingoid base coating was stable at 72 h in saliva (but not in blood); in fact, additional sphingosine from saliva adhered to the coating during incubation so that the layer was thicker when viewed under scanning electron microscopy. Endotracheal tubes that were coated with sphingosine or phytosphingosine, dried, and stored, retained the same level of bactericidal activity for at least 7 days. 

Beck and colleagues [90] applied a sphingosine coating on titanium, commonly used for orthopedic implants. This treatment was 99.999% successful in preventing *S. epidermidis* biofilm growth even when immersed in a bacterial culture. Sphingosine treatment of *S. epidermis* biofilms killed 99.942% of the bacteria. This group tested three different *S. epidermidis* strains, including one oxacillin-resistant strain, and one methicillin-resistant strain. Scanning electron microscopy confirmed that cells of treated biofilms were damaged and distorted, similar to studies completed by Fischer [36] and Bibel [80,82]. The sphingosine coating on titanium pieces was stable when immersed in culture media for 26 h and when implanted, ex vivo, into murine femur bones, indicating that sphingosine might be useful to prevent early periprosthetic infections. Additionally, because sphingosine-1-phosphate (a phosphorylated version of sphingosine) is important in bone growth [91], sphingosine treatment may have additional positive effects on healing. This study is also significant because of the relative paucity of antimicrobial or bactericidal agents that are effective against biofilms. 

A later study further developed the idea of clinical use of sphingosine as an anti-bacterial drug by demonstrating that sphingosine inhalation by Goettingen mini pigs has no adverse side effects [92]. Pigs inhaled sphingosine, suspended in a physiologic saline with a surfactant, 1–2 times per day for two weeks before being extensively tested for adverse health effects. Blood tests, morphological tests, immunohistochemistry, and gross examination demonstrated that bronchial cells of the lungs accumulated sphingosine with no adverse effects to bronchial epithelial cell integrity and no influx of leukocytes. 

## 5. AML Lipid Mechanisms

Relatively little is known about specific mechanisms of AML activity. Early studies proposed a lipophilic activity that would permit adsorption to the cell surface. This proposal was based on evidence that the chemical properties of the most active lipids, such as lauric acid, allowed an optimum balance of water solubility and lipophilic activity [93]. General trends from subsequent research demonstrated that antimicrobial activity is a function of carbon length as well as the presence, number, and orientation of double bonds [94], leading to the proposal that mechanisms are likely complicated and could include different mechanisms for different bacteria/lipid combinations.

Generally, most studies above reported that treatment of bacteria with AMPs caused creation of pores in the bacterial cell membrane and/or lysis of cells, which indicates a potential disruption of various cellular processes either by interference of spatial arrangement or by direct binding to proteins [94]. Based on demonstrated differential activity of sphingoid bases and fatty acids against various bacteria, it is likely not a basic surfactant effect. Desbois and Smith [94] published an extensive review of proposed fatty acid mechanisms, noting that the cell membrane is likely the prime target, which, if interrupted, could interrupt cellular respiration. Lipids may be transported into the cell and accumulate in the cytoplasm where they can potentially interact with various protein components of the cytosol, inhibiting cytosolic enzymes or bacterial fatty acid synthesis. Lipids may also insert into the membranes of bacteria causing changes in the physical properties of the membrane (e.g., membrane fluidity, size, shape) which could potentially disrupt energy production through spatial orientation of electron transport chain components [94]. Yoon et al. [95] discussed this further, naming destabilization of the bacterial membrane as the main mechanism of fatty acid activity against bacteria.

Several studies support membrane disruption and/or activity against specific cell wall components. Parsons et al. [96] also demonstrated rapid *S. aureus* membrane depolarization and disruption of metabolism when treated with fatty acids, but took their study further, reporting that even inactive fatty acids were active against *S. aureus* strains whose cell walls were deficient in teichoic acids. This supports the idea of structure-specific fatty acid toxicity. Likewise, Bibel et al. [82] used electron microscopic studies to demonstrate that L-forms of *S. aureus,* lacking cell walls, were relatively resistant to the lethal effects of sphinganine. Treatment of *Helicobacter pylori* with oleic or linoleic acid also induces disruption of the cellular membrane, inducing cell lysis [97]. Other studies by Fischer et al. [36,37] demonstrated that AML-treated *P. gingivalis, S. aureus,* and *E. coli* exhibited lysing of cell membranes and flocculation of intracellular contents to form bacterial inclusions. Additionally, these studies demonstrated that at least some of the treatment lipids were retained within the membranes of *P. gingivalis* [37].

Recent studies demonstrate differential protein regulation in *P. gingivalis* when treated with sapienic acid [37,59]. Treatment of *P. gingivalis* with sapienic acid produced statistically significant overrepresentation of proteins involved in respiration, metabolism, energy production, iron acquisition and processing, and lipid biosynthesis, when compared to healthy, untreated bacteria. Further studies by this group suggested that along with the expected increase in proteins related to energy production, iron acquisition and processing, and respiration, and the decrease in some virulence proteins, sapienic acid treated *P. gingivalis* also showed an increase in proteins related to lipid metabolism [59]. Because endogenous *P. gingivalis* cell membrane phospholipids contain branched fatty acids of approximately the same length as sapienic acid, it may be possible for sapienic acid to be incorporated into newly synthesized *P. gingivalis* phospholipids, which would alter the fluidity of the membrane. This study suggests that while trying to overcome excessive sapienic acid uptake, *P. gingivalis* increased energy production and fatty acid biosynthesis, possibly to compete with insertion of toxic fatty acid species into the bacterial plasma membrane. Increased fatty acid biosynthesis is supported by evidence of preferential serine metabolism, which is an important intermediate for sphingoid bases present in *P. gingivalis* membranes [98,99,100], and significant increases in FabH and FabF, proteins associated with the FAS-II fatty acid biosynthesis pathway [59].

Several studies suggest that fatty acid biosynthesis may be inhibited by treatment with some antimicrobial fatty acids. A study by Zheng and colleagues [101] isolated several clinical bacterial strains and treated them with medium chain fatty acids, including linoleic acid, palmitoleic acid, oleic acid, and arachidonic acid. All of the unsaturated fatty acids tested, but none of the saturated fatty acids, induced inhibition of the fatty acid synthase protein, FabI. Treatment of *S. aureus* with capric, lauric (both saturated), and α-linoleic acids (polyunsaturated) also inhibited fatty acid biosynthesis in *S. aureus* [102]. In this study, labelled acetate, used as a carbon source, was incorporated into fatty acids in *S. aureus* membrane phospholipids at different rates, depending on the treatment and pH, which effectively demonstrated the differential effects of lipid treatments.

Unsaturated fatty acids are generally more potent that saturated fatty acids of the same length [47,101]. Maximum activity of saturated fatty acids is found in short fatty acids 12-carbons in length [6]; however, some monounsaturated fatty acids of 14–16 carbons have similar antimicrobial activity [6,48]. Additionally, some shorter chain fatty acids exhibit potent antifungal activity [45,46,103]. Sapienic acid, one of the most active AMLs, is a 16-carbon with a cis double bond at carbon 6, which causes a twist in the molecule, shrinking it to roughly the same physical size as a 14-carbon fatty acid; however, the 18-carbon sebaceous sapienic acid does not exhibit antimicrobial activity [15].

Activities of AMLs described here are similar to numerous other host innate immune factors, including anionic peptides [104], cathelicidins [105], and defensins [106,107], inducing extensive damage to gram-positive and gram-negative bacteria similar to what I have described here, including, including flocculation of intracellular contents, alteration of bacterial cytoplasmic membranes, and inhibition of various cellular processes [108]. AMLs have also demonstrate synergistic activity with other antimicrobial products. Preliminary research demonstrates synergistic effects of sphingoid bases with cathelicidin and LL37 against a range of Gram positive bacteria, Gram negative bacteria, and yeast [109]. Lauric acid has been shown to enhance chlorhexidine activity against *S. mutans* [110]. A combination of two AMLs, phytosphingosine and N-lauryl-arginine ethyl ester laurate, appear to act synergistically, as the combination is more effective against several coryneform species than either of the lipids individually [111], lending further credence to multiple endogenous lipids acting as part of host innate immunity.

## 6. Consequences of Endogenous AML Disruptions

Perturbations of endogenous lipid levels can induce pathogenesis due to increased microbial colonization. Multiple studies highlight the implications of disrupted endogenous lipid levels. For example, both decreased levels of sphingosine [112] and deficient sapienic acid production [113] are associated with atopic dermatitis and contribute to increased *S. aureus* skin colonization in otherwise healthy individuals. The mechanism of sapienic acid deficiency in atopic dermatitis is unknown, but is likely reflective of the altered skin microbiome, based on the finding that sebaceous triglycerides are hydrolyzed by microbial lipases [25]. The underlying mechanism of deficient free sphingoid bases in atopic dermatitis is quite unusual. An enzyme that is not expressed in healthy epidermis, glucosylceramide sphingomyelin deacylase, is expressed in the skin of patients with atopic dermatitis [114]. Glucosylceramide sphingomyelin deacylase hydrolyzes the amide linkage of sphingomyelin and glucosylceramide, yielding fatty acids, 1-O-glucosylsphingosines and 1-O-phosphocholines [115]. These sphingosine derivatives are not hydrolyzed to yield free sphingoid bases but the action of glucosylceramide sphingomyelin deacylase does alter the ceramide content and composition of the stratum corneum. A later discovery of sphingomyelin deacylase, which demonstrated three-to-five times more activity in atopic dermatitis patients [116], helps explain the relative lack of sphingosine. Sphingomyelin deacylase catalyzes the hydrolysis of sphingomyelin to sphingosylphosphorylcholine (instead of ceramides) contributing to a reduction in ceramide production in these patients [117]. 

Cystic fibrosis has also been linked with deficiencies of several AMLs. Patients with cystic fibrosis are highly susceptible to serious infections by multiple opportunistic bacterial species, including *P. aeruginosa, S. aureus, Haemophilus influenza,* and others. Pewzner-Jung and colleagues [118] demonstrated that deficient ceramidase activity correlated with surface expression of β1 integrins [119] and a subsequent reduction of sphingosine concentrations on the luminal surfaces of tracheal and bronchial endothelial cells of cystic fibrosis patients and mice. This group also demonstrated that treatment with ceramidase or inhalation of sphingosine reverses that condition in cystic fibrosis mice, preventing severe infection even when mice were directly inoculated [118]. Importantly, inhalation treatments with sphingosine resulted in reduced inflammatory cytokines, indicating a reduction in inflammation as well as reduction of bacterial loads, and no histological damage was present after extended sphingosine inhalation. Cystic fibrosis has also been linked to a deficiency in fatty acid production that creates an imbalance fatty acid concentrations [120,121,122]. In one recent study, cystic fibrosis patients had a significantly increased ratio of arachidonic to docosahexaenoic acid. In yet another study, innate immunodeficient mice were unable to clear *S. aureus* or *Streptococcus pyogenes* infections, which was linked to mutation of an enzyme necessary for palmitic and oleic acid production [123]. 

It is reasonable to assume that defective production of lipids, or imbalances in lipid ratios could be responsible for other skin and oral diseases. Epidermal carriage status of *S. aureus* is relatively stable within individuals - especially in the nose [124], which is also rich in antimicrobial peptides [125] and AML groups [12]. Nasal fluids from *S. aureus* carriers do not kill *S. aureus*, while nasal fluids from non-*S. aureus* carriers are active against *S. aureus* [126]. These studies suggest that AMLs may contribute to innate defense mechanisms by protecting against colonization of the skin by *S. aureus;* in fact, an individual’s bacterial flora may even be partially shaped by their specific lipid ratios.

## 7. Summary and Conclusions

AML roles in the maintenance of health are emerging and multiple studies suggest that these lipids actively work alongside AMPs and other innate factors to help maintain overall health. Free fatty acids derived from human sebaceous triglycerides, especially lauric acid and sapienic acid, are potent antimicrobials found on human skin and mucosal surfaces. Undecylenic acid, present in sweat, is a potent antifungal that is commonly used in over-the-counter compounds. The epidermally derived sphingoid bases, sphingosine, dihydrosphingosine and 6-hydroxysphingosine, are also potent and broad-acting antimicrobials. These, along with other natural AMLs, act as part of the innate immune system of skin, mucosal membranes, and various body fluids. 

More exploratory studies for the use of AMLs in technology development and as therapeutic treatments are also emerging. Important features of the development of AML-containing therapeutic or prophylactic treatments include the relative lack of side effects, the tendency of AMLs to reduce inflammation, and the ability of AMLs to prevent and treat biofilms. Because the AMLs discussed in this review are endogenous lipids, the risk of side effects with use is very low. Multiple studies discussed in this review demonstrated a lack of side effects with treatment. AMLs have been tested as treatments for burn patients, cystic fibrosis patients, and as preventative treatments on plastic and metal implant materials. Most of these studies included histological examination of test animals, immunohistochemistry, blood tests, and other tests to confirm that tissues and cells were undamaged after repeated treatments. Many studies also included cytokine studies, demonstrating decreased inflammation with the use of AMLs in treatments. Finally, multiple studies discussed here demonstrate AML activity against biofilms, which is desperately needed as many antibiotics are generally ineffective against biofilms. 

Fatty acids are being incorporated into various therapeutic products such as Desinex^®^, an antifungal cream containing undecylenic acid (produced commercially by distillation of castor bean oil) as its active ingredient [30]. Lauric acid is so widely used that it is readily available online and found in a myriad of cleaning and personal healthcare products. Undecylenic acid added to denture liners inhibits normal growth cycles of *C. albicans*, thus inhibiting its proliferation [72]. A 15%–20% solution of undecylenic acid also decreases viral shedding and pain in patients with herpes simplex viruses [127,128]. Sphingosine [129], phytosphingosine [129], and lauric acid [61] have also been explored as therapeutic options for acne treatment. 

The dose-dependent and specific antimicrobial activity exhibited by various AMLs supports full recognition of AMLs as a critical part of innate immune function. Generally, fatty acids and sphingoid bases differentially kill bacteria, viruses, and fungi in a dose dependent manner, often inducing visible intracellular and extracellular damage similar to that seen with various antimicrobial peptides. Although mechanisms of action are still relatively unknown, possibility partially due to the difficulties of working with lipids, several studies have begun to outline mechanisms of AML activity against microorganisms. However, the broad range of AML activity reported in this review supports the idea that AMLs are both selective and specific in their activity against microorganisms, suggesting that we may find varied mechanisms of action specific to AML-pathogen pairs based on the structural characteristics of each. We still have much to learn about this newly described class of innate molecules, and we expect to learn more in the next few decades.

## Figures and Tables

**Table 1:** Composition of neutral lipids from saliva [15] and the mucosal surface [31]. These unpublished data are used with permission from Dr. Philip Wertz. Lipid samples from the mucosal surface were obtained using Whatman filter paper held against the cheek for one approximately minute. Squalene and wax esters are biochemical markers of human sebum.

Lipids	µg/mL Saliva	µg/cm^2^ Surface
Squalene	2.0	13.1
Cholesterol esters	0.2	1.1
Wax esters	1.7	9.4
Triglycerides	26.4	21.8
Fatty acids	2.0	7.8
Cholesterol	10.0	15.0
Total	42.3	68.2

**Table 2:** Innate free fatty acids exhibit antimicrobial activity against a variety of opportunistic bacteria, viruses, and fungi. Lauric acid is the most broadly antimicrobial fatty acid of those naturally occurring on the skin, mucosal membranes, and in sweat. These data are summarized from multiple studies; however, huge variability in reporting of AML activity precludes the compilation of detailed concentrations into a single chart. Readers are referred to individual citations for specific concentration details.

Microbe	Sapienic Acid	Lauric Acid	Undecylenic Acid
*Aggregatibacter* *actinomycetemcomitans*	[38]	[38]	
*Candida albicans*		[47,56]	[30,72]
*Clostridium difficile*		[68]	
*Epidermophyton inguinale*			[30]
*Escherichia coli*		[4,63]	
*Fusobacterium nucleatum*	[38]	[38]	
*Helicobacter pylori*		[73,74]	
*Herpes simplex virus 1 and 2*		[4,71]	[30]
Micrococci		[47]	
*Microsporum audouinii*		[54]	[30]
*Nocardia asteroides*		[47]	
Pneumococci		[47]	
*Porphyromonas gingivalis*	[37]	[37]	
*Propionibacterium acnes*		[55,61,74]	
*Respiratory syncytial virus*		[4]	
*Shigella sonnei*		[75]	
*Staphylococcus aureus*		[38,47,55,63,76]	
*Staphylococcus epidermidis*		[4,47,55,76]	
Streptococci		[4,42,47,57,77]	
*Streptococcus mitis*	[38]	[38]	
*Streptococcus mutans*		[58]	
*Streptococcus sanguinis*	[38]	[38]	
*Trichophyton rubrum*			[30]
*Trichophyton mentagrophytes*			[30]
*Vaccinia virus*		[4]	

**Table 3:** Innate free sphingoid bases, naturally occurring on skin and mucosal membranes, exhibit antimicrobial activity against a variety of opportunistic bacteria, viruses, and fungi. These data are summarized from multiple studies; however, huge variability in reporting of AML activity precludes the compilation of detailed concentrations into a single chart. Readers are referred to individual citations for specific concentration details.

Microbe	Sphingosine	Dihydrosphingosine (Sphinganine)	6-hydroxy- Sphingosine
*Acinetobacter baumannii*	[89]	[89]	
*Acinetobacter lwoffii*		[82]	
*Bacillus subtilis*		[82]	
*Candida albicans*	[82]	[80,82,83]	
Corynebacteria	[38]	[38]	
*Epidermophyton floccosum*	[82]		
*Escherichia coli*	[38]	[38,80]	
*Fusobacterium nucleatum*	[38]	[38]	
*Micrococcus luteus*		[80]	
*Neisseria meningitides*		[82]	
*Porphyromonas gingivalis*	[37]	[37]	[37]
*Propionibacterium acnes*	[40]	[40,80]	
*Pseudomonas aeruginosa*	[89]	[80,89]	
*Serratia marcescens*		[80]	
*Staphylococcus aureus*	[80,89,90]	[80,81,82,83,89,90]	[80]
*Staphylococcus epidermidis*	[38,90]	[38,80]	
*Streptococcus mitis*	[38]	[38,81]	
*Streptococcus sanguinis*	[38]	[38]	
*Streptococcus pyogenes*		[80]	
*Trichophyton mentagrophytes*	[82]		
*Trichophyton tonsurans*	[82]		
